# Shifting Rhythms: A Systematic Review Exploring the Multifaceted Effects of Shift Work and Circadian Disruption on Employee Cardiovascular Health

**DOI:** 10.7759/cureus.71003

**Published:** 2024-10-07

**Authors:** Ayesha Hanif, Donatus K Okafor, Gitika Katyal, Gursharan Kaur, Hafsa Ashraf, Adiprasad Bodapati, Tuheen Sankar Nath

**Affiliations:** 1 Internal Medicine, Fatima Jinnah Medical University, Lahore, PAK; 2 Internal Medicine, California Institute of Behavioral Neurosciences & Psychology, Fairfield, USA; 3 Internal Medicine, Shalamar Medical and Dental College, Lahore, PAK; 4 Surgical Oncology, Tata Medical Center, Kolkata, IND

**Keywords:** cardiovascular disease, circadian rhythm, healthcare worker safety, inverted circadian rhythm, ischemic heart disease, night shift, nurses, occupational health and safety, risk factors cardiovascular diseases, shift work schedule

## Abstract

Shift work has long been studied as a contributing risk factor for cardiovascular disease. This study aims to provide a comprehensive summary of data regarding shiftwork and its impact on the cardiovascular system from the last decade. It explores the association of shift schedules with multiple aspects of cardiovascular disease and the physiological processes that lead up to it. It also identifies gaps in current knowledge regarding the topic. Two hundred and sixty-eight articles were gathered from PubMed, Google Scholar, and Science Direct using relevant medical subject headings (MeSH) strategy and advanced search using keywords including ‘Shift work,’ ‘Night shift,’ ‘Occupational health,’ ‘Circadian rhythm,’ ‘Cardiovascular disease,’ ‘Cardiovascular health.’ The search was conducted in April and completed in May 2023. Systemic reviews, meta-analysis, cohort and cross-sectional studies from the last 10 years were included, and assessment of multiple systematic reviews (AMSTAR), Newcastle Ottawa, and Joanna Briggs Institute (JBI) tools were used, respectively, for quality assessment. A total of 14 articles were included in our review, including five systematic reviews and meta-analyses, six prospective cohort studies, and three cross-sectional studies. Each study reported a significant association between shift work with some aspect of cardiovascular disease. An increase in the risk of myocardial infarction, coronary heart disease, hypertension, atherosclerosis, and metabolic syndrome is reported. Circadian disruption, unhealthy diet, and emotional and physiological stress contribute to these effects. Oxidative damage and inflammatory biomarkers appear to play a role in this process, but more research is warranted for a deeper understanding of these changes. Despite an abundance of evidence pointing towards the short-term and long-term harm to shift workers' cardiovascular health, there is limited research regarding the policies that are needed to better monitor cardiovascular damage in employees. The focus needs to shift toward prevention-based policies and their efficacy in workplace settings.

## Introduction and background

Whether it's access to healthcare services, purchasing a product, or receiving customer support, in today's fast-paced society, there is an increasingly prevalent need for efficient, round-the-clock service. Shift work is just one of the many strategies that businesses and industries have relied upon to match consumer expectations. Employees work in 'fixed shifts', i.e., nontraditional working hours for extended periods of time, or 'rotating shifts' where work schedules periodically rotate between different shifts (night, evening, or morning), typically spanning a week or a month.

While this helps institutions provide continuity in service, it does so at a huge cost to the workers' well-being. Not only do shift workers report lower job satisfaction, but they also suffer from poor sleep, exhaustion, and burnout [1]. Over the years, research has linked shift work to various mental health problems, cardiometabolic disorders, and even cancer [2].

Numerous epidemiological studies and scientific investigations have explored the impact of shift work on the cardiovascular health of healthcare personnel. The growing body of evidence suggests that shift work may contribute to an increased risk of developing cardiovascular disease (CVD), encompassing conditions such as hypertension, myocardial infarction (MI), stroke, and other adverse cardiac events [3,4]. Understanding the relationship between shift work and CVD among healthcare professionals is crucial for identifying preventive strategies and implementing appropriate interventions to safeguard the health of this vital workforce.

Shift work disrupts the circadian rhythm [5], the internal body clock that regulates various physiological processes, including sleep-wake cycles, hormone secretion, and metabolism [6-8]. Moreover, it involves exposure to various occupational stressors, including high workloads, emotional demands, and unhealthy dietary habits [9]. These factors, combined with disrupted sleep patterns, may lead to dysregulation of cardiovascular and metabolic functions, thereby increasing the vulnerability to cardiovascular diseases among healthcare professionals.

While several individual studies have investigated the relationship between shift work and CVD risk [2-4], a comprehensive synthesis of the existing evidence is needed to provide a more robust understanding of the topic. This systematic review aims to contribute to the existing knowledge by providing a comprehensive evaluation of literature from the last decade, identifying research gaps, and generating evidence-based recommendations to mitigate the adverse cardiovascular effects of shift work among healthcare personnel.

## Review

Methods

A systematic review of the literature following the Preferred Reporting Items for Systematic Reviews and Meta-Analysis (PRISMA) model was conducted by two independent authors for the association of shift work with cardiovascular disease. The following databases were used: PubMed, Google Scholar, and Science Direct. Only studies from 2013 and onward were included. The search was concluded in May 2023. The terms used in the searches were shift work/cardiovascular disease/night shift/healthcare personnel/occupational health. A relevant MeSH strategy was created for results on PubMed: (("Work Schedule Tolerance"(Mesh)) OR ("Shift Work Schedule"(Mesh)) OR ("Night Work"(Mesh)) OR ("Workload"(Mesh))) AND (("Cardiovascular Diseases"(Mesh)) OR ("Myocardial Infarction"(Mesh)) OR ("Stroke"(Mesh)) OR ("Heart Diseases"(Mesh)) OR ("Hypertension"(Mesh))) AND (("Health Personnel"(Mesh)) OR ("Occupational Health"(Mesh)) OR ("Occupational Exposure"(Mesh)) OR ("Occupational Diseases"(Mesh))). An assessment of the references of the included studies and a search of their citations in the PubMed database was performed to identify any additional studies. A third author double-checked for duplicates and possible errors.

**Figure 1:**
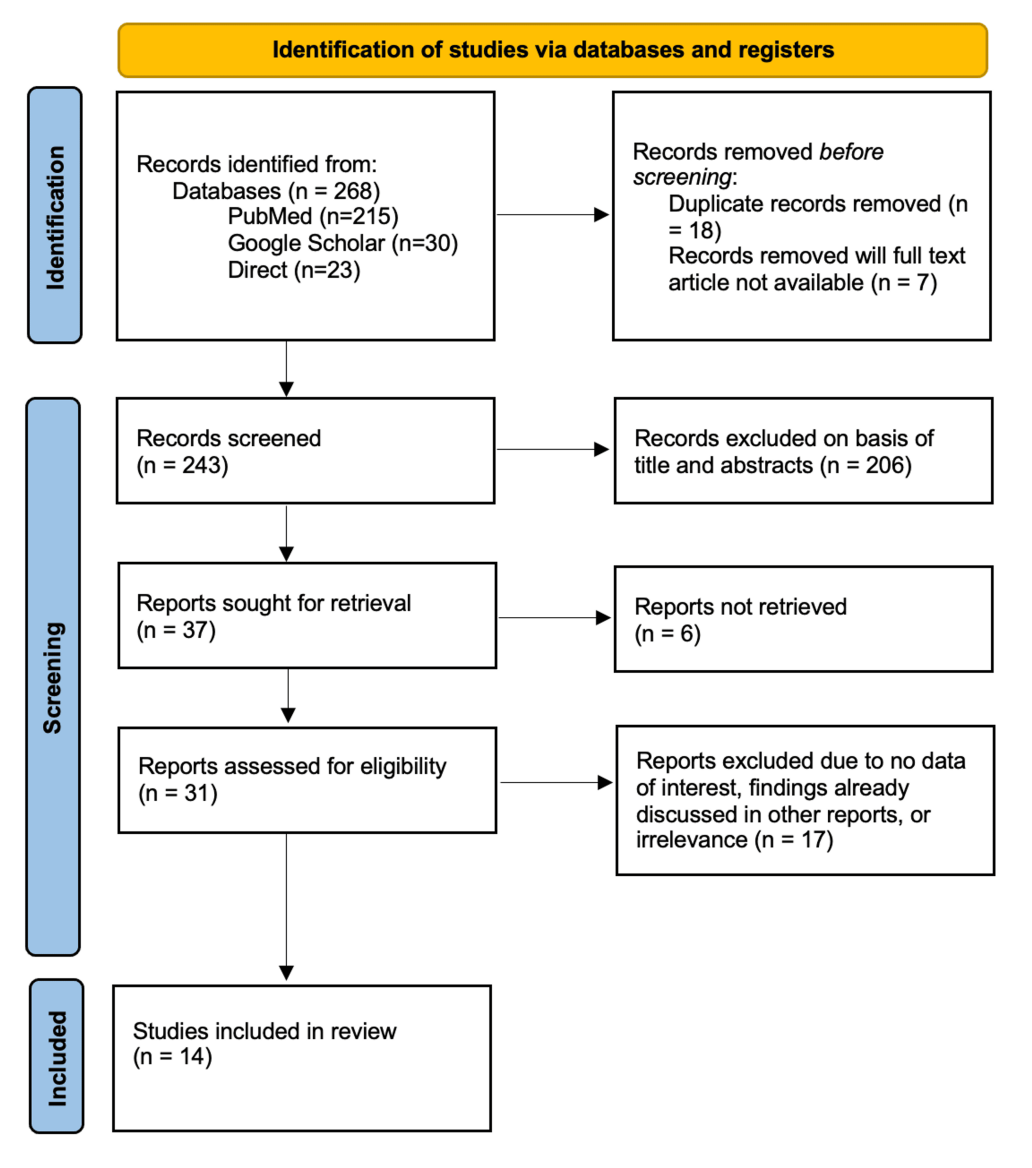
PRISMA flow diagram for systematic reviews. PRISMA: Preferred Reporting Items for Systematic Reviews and Meta-Analysis. Reference: [10].

The study topic was framed in accordance with the population, intervention, comparison, and outcome (PICO) framework. The process of selecting the research papers via the PRISMA flowchart is depicted in Figure 1.

Studies were eligible for inclusion if they: (1) Assess cardiovascular disease outcomes, including myocardial infarction, stroke, hypertension, coronary artery disease, arrhythmia, or other cardiovascular conditions; (2) were conducted on human participants; (3) studies that involve various types of shift work schedules (e.g., night shifts, rotating shifts, and irregular shifts) as the primary variable; (4) studies published in the last ten years to ensure the review includes recent research; and (5) were published in English language. Studies were excluded that (1) only assess the impact of shift work on non-cardiovascular outcomes; (2) studies that lack sufficient data or statistical analysis related to the association between shift work and cardiovascular disease; and (3) studies published before the defined timeframe (e.g., more than ten years ago).

Data Extraction

The following information was collected from each study: author surname, year or publication, study design, study population, aim, exposure, means of exposure assessment (when applicable), exclusion criterion (when applicable), follow-up period (when applicable), outcome measures, and results.

Results

Our search returned 268 articles, of which 215 were from PubMed, 30 from Google Scholar, and 14 from Science Direct. Of these 14 relevant articles met the aforementioned inclusion criterion and were therefore analyzed. Our review included two systematic reviews, three systematic reviews with meta-analysis, six prospective cohort studies, and three cross-sectional studies.

Six out of 14 studies include participants from the general population, while the rest eight cater mostly to healthcare workers. Out of these eight, five have studied nurses exclusively. A systematic review by Rosa and colleagues [2] provides a comprehensive outlook on the many ways shiftwork and its associated de-synchronization of circadian rhythm affects the health of nurses. It proposes that six years of shift work may increase the risk of developing an MI by as much as 30%, along with a higher risk of arteriosclerosis as well [11]. A meta-analysis conducted in the same year [2] further added that each additional year of shift work increases the risk of ischemic heart disease (IHD) by 0.9%. A dose-response association was observed between the duration of shit work and pooled relative risk of IHD (P = 1.009, 95% CI: 1.006-1.012).

Vetter et al. [12] studied a similar clinical outcome, i.e., coronary disease and its manifestations, such as fatal and non-fatal myocardial infarction, coronary artery bypass grafting (CABG), and angioplasty. Rotating night shift data and cumulative exposure to shift data from two large cohorts from the Nurse's Health Database (NHS). NHS 1 and NHS 2 (NHS (1988-2012): N = 73,623, and NHS2 (1989-2013): N = 115,535) were analyzed. They found that there was a small but statistically significant increase in the incidence of coronary heart disease (CHD) in both cohorts. Also, the mean ages in NHS1 and NHS2 were 54.5 years and 34.8 years, respectively. It was observed that initially, women who worked <five years of shift work did not have a significantly increased CHD risk in age-adjusted analysis when compared to women without a shift work history. However, after five or more years of shift work, even after multivariate analysis, the elevated risk persisted in NHS1 and NHS2 for >10 years of shift work. This shows an important relation between shift work with duration in years. They also noted that this risk waned as more time passed after quitting shift work.

Another study [13] has reported that individuals who have been exposed to night work are at a potentially higher risk of cardiovascular mortality, as well as all-cause mortality. This higher risk of all-cause mortality also appears to be driven mainly by cardiovascular causes. A similar study focusing specifically on the nursing population reports a similar conclusion. Women who worked rotating night shifts for six to 14 years and 15 or more years had a higher risk of mortality from ischemic heart disease (IHD) [14]. The hazard ratios (HRs) for these groups were 1.22 (95% confidence interval (CI): 1.02-1.46) and 1.31 (95% CI: 1.09-1.59), respectively.

A Danish study done on healthcare workers corroborates this association. They found that there is an elevated risk of ischemic heart disease (IHD) associated with an increase in the number of night shifts and consecutive spells of night shifts [15]. Specifically, the risk of IHD increased by 3% for every additional 10 night shifts (HR for beta 1.03, 95% CI: 1.00-1.05) and by 6% for every additional 10 spells of ≥three consecutive night shifts. This study also set out to examine whether atrial fibrillation, which is a leading risk factor for ischemic stroke [16,17], was more common in employees working night shifts. Interestingly, no significant association was found, despite earlier studies [16,18] suggesting otherwise. Vetter and colleagues also claimed that the increased risk of CHD is present even in the absence of other mediators such as hypertension, diabetes, and hypercholesterolemia. Another study from the UK Biobank [19] sheds further light on the role of mediating factors on incident CVD. They found that more than half (52.3%) of the association between CVD and shift work can be explained by mediators such as current smoking status, sleep duration, adiposity, and other biomarkers such as glycated hemoglobin (Hba1c), cystatin C, and gamma-glutamyl transferase (GGT) that reflect on diabetes, kidney and liver function related to alcohol, respectively.

The multifactorial effect of shiftwork on the cardiovascular system (CVS) warrants in-depth study on the pathways that lead from shiftwork-related chrono-disruption to overt cardiovascular disease. The majority of the research so far has been consistent in their view that this detriment to cardiac health is due to a web of interlinked physiological mechanisms and psychosocial behaviors [1,20-22]. Evidence suggests that shift workers, night shift in particular, are more likely to indulge in unhealthy eating habits [21,23], consume increased amounts of caffeine [24,25], suffer from insufficient sleep [26], and be in states of relatively high physical and emotional stress over the night. These behaviors then translate to the body releasing increased amounts of catecholamines [7,27,28] and inflammatory biomarkers [29,30]. They are also associated with a concurrent decrease in melatonin secretion in night shift workers [31], which has otherwise been shown to have a favorable effect on glucose and lipid metabolism, besides its role as a blood pressure regulator and anti-oxidant [32]. Gibson [33] further examined the relation of oxidative stress in shift workers. She found elevated levels of reactive oxygen species, DNA damage, lipid peroxidation, and DNA repair capacity in healthcare personnel who work shifts. She noticed this difference not just in comparison between a single night shift and a single day shift but also observed it when comparing regular night shift workers to employees who had never been involved in shift work. Bhatti et al. supported these findings and linked melatonin suppression to the aforementioned changes in oxidative stress [34].

Other researchers have similarly tried to quantify the changes at a biochemical level that precede the clinical manifestation of CVD. Atherosclerosis is one such process. Jankowiak et al. [35] studied a large cohort (the Gutenberg Health Study, N = 15,010) from 2007-2010 and used the data to examine three main study outcomes that included measurements of arterial stiffness, vascular function (reactive hyperemia (RH) index), and intima media thickness (IMT). They found that workers who had been involved in more than 660 night shifts within the last decade had increased arterial stiffness of 0.33 m/s as compared to non-night workers. People who had a higher cumulative night shift experience also had a decreased vascular function by −0.054 RH index points. However, a significant difference between intimal medial thicknesses was not found. This differs from Rizza et al.'s findings, who found significantly higher carotid intimal medial thickness (cIMT) in former and rotating night shift workers (p = 0.023) [29] when compared to day workers. Going a step further, they theorize that interleukin-1B mediated abundant release of interleukin-6 might be behind the inflammation leading to the atherosclerotic process. They derive the data regarding this inflammatory cascade mainly from animal studies, but the clinically significant outcomes seem promising and warrant further investigation.

Last but not least, hypertension has also been looked at as one of the outcomes of prolonged shift work [36-38]. More specifically, a large cross-sectional study based on nurses from the Hebei province in China showed that those who worked five to 10 or greater night shifts in the last six months had a higher risk of hypertension [4]. A small yet significant increase in their odds ratio (OR) values was observed with increasing night shift frequency. They also investigated how the age of the nurses factored into this relationship. Not surprisingly, it was found that age and night shift frequency have an additive effect on the prevalence of hypertension. When compared with younger nurses (18-25 years) with no night shift, those in the 36-45 and 46-65 age brackets with >10 nights per month in the last six months had an OR value of 3.430 (95% CI: 2.273‐5.175) and 7.398 (95% CI: 5.595‐9.781), respectively. Additionally, women in the latter group are already at risk for peri-menopause/menopause-associated CVS changes and have been exposed to more number of shift work years on account of age and seniority. Shift workers are also twice as likely to develop metabolic syndrome, which is a major precursor to cardiovascular disease and mortality [39]. Sooriyaarachchi et al. [40] recently found this association in their systematic review and meta-analysis, where 10 out of 12 studies linked an increased risk of metabolic syndrome to shift work. The pooled OR of metabolic syndrome based on all twelve studies was 2.17 (95% CI: 1.31-3.60, P = 0.003; I2 = 82%, P < 0.001). They focused exclusively on healthcare workers and found a stronger association when compared to previous studies [41-43] on non-healthcare populations.

Also, individuals who are already hypertensive are more likely to transition to cardiometabolic multi-morbidity (CMM), i.e., the presence of two or more cardiometabolic diseases at the same time, which is an increasingly common problem in today’s time [44]. Using data from the United Kingdom (UK) biobank and a large study population, Yang et al. studied the interplay between participants’ chronotype (morning/evening preference), night shift frequency and duration and their influence on CMM [45]. The study focused on participants with hypertension and found that specific combinations of factors were linked to an increased CMM risk. Morning chronotype combined with more than 10 night shifts per month resulted in a 26% higher risk of CMM. Similarly, more than 10 night shifts per month combined with either < seven or > eight hours of sleep increased the risk by 43%. Morning chronotype with ≤ 10 night shifts per month led to a 22% higher risk, while ≤10 night shifts per month combined with <seven or >eight hours of sleep resulted in a 31% higher risk. Lastly, working night shifts for ≤10 years with <seven or >eight hours of sleep showed a 24% higher CMM risk. Like other studies [4,46], this study also confirms that increased shift frequency and lower sleep levels, especially in morning chronotypes, contribute to association with CMM. 

A summary of the primary study characteristics extracted from the aforementioned studies can be found in Table 1. 

**Table 1:** Study characteristics. N/A: not available; RR: relative risk; CI: confidence interval; IHD: ischemic heart disease; CVD: cardiovascular disease; CVS: cardiovascular system; MetS: metabolic syndrome; r-NSW: rotating night shift work; f-NSW: former night shift work; DW: day worker; CIMT: carotid intima media thickness; NHS: Nurse Health Database; AF: atrial fibrillation; CMM: cardiometabolic mortality.

Study ID	Author	Year	Study design	Population	Sample size (n)/number of articles	Aim	Exposure	Exposure assessment	Exclusion criterion for the study population	Follow-up	Outcome measures	Result
1	Rosa et al. [2]	2019	Systematic review	Nurses (mostly white female)	24 Articles	To describe the effects of shift work and desynchronization of circadian rhythms on nurse’s health.	Shift work	N/A	N/A	N/A	Various aspects of health, cardiovascular and otherwise.	Shift work, risk factors for stress, sleep disorders, metabolic disorders, diabetes, cardiovascular disorders, and breast cancer.
2.	Cheng et al. [3]	2019	Systematic review and meta-analysis	Nonspecific	n = 320,002	To conduct a systematic review and meta-analysis of epidemiological evidence and summarize the potential relationship between shift work and IHD.	Different types of shift work	N/A	N/A	N/A	Risk or incidence of IHD	Positive association (RR 1.13; 95% CI: 1.08–1.20) between shift work and IHD.
3.	Su et al. [13]	2021	Meta-analysis of cohort studies	Various cohort participants	n = 958,674	Conducting a meta-analysis by summarizing the most up-to-date evidence to systematically evaluate the association between shift work or night work and risk of total deaths, as well as deaths due to CVD and cancer.	Any type of shift work	N/A	N/A	N/A	All-cause mortality, mortality due to CVS or cancer.	Night shift work: high risk of CVS-related mortality
4.	Madeline Gibson et al. [33]	2021	Systematic review	Mostly healthcare persons	n = 1796; F: 1077, M: 719	To contribute to a consensus on the impact of circadian rhythm disruption from night shift work on oxidative stress-identify potential protective factors, areas for further research and promote the long-term health of shift workers.	Night shift and day shift (regular or rotational)	N/A	N/A	N/A	Levels of oxidative stress indicators.	Night shift significantly high in oxidative stress.
5.	Sooriyaarachchi et al. [40]	2022	Systematic review and meta-analysis	Shift workers in the health sector	n = 3697	To summarize evidence on the association between the risk of developing MetS and shift work among employees of healthcare services.	Different types of shift work	N/A	N/A	N/A	Prevalence/incidence of MetS in shift workers	Shift workers exhibited more than a twofold increase in the chance of developing MetS in comparison with day workers.
6.	Rizza et al. [29]	2020	Cross-sectional study	Hospital employees	n = 187 (r-NSW, n = 88), (f-NSW, n = 35), (DW, n =64)	Investigate the association between night shift work, inflammation and carotid intimal medial thickness.	Rotating night shift, former night shift work vs day workers.	Standardized interviews. Outcome assessment: lab tests, carotid u/s	Presence of diabetes, liver disease, renal insufficiency, heart failure, coagulopathy, or any other severe systemic disease.	N/A	CIMT	A possible link bridging night shift work, inflammation and carotid intimal medial thickness.
7.	Vetter et al. [12]	2016	Prospective cohort study	Female registered nurses	n = 189,158 NHS (n = 73,623), NHS2 (n = 115,535)	Determine whether rotating night shift work is associated with CHD risk.	Rotating night shift work (≥ three night shifts/month, plus day and evening shifts)	Questionnaires at baseline, biennial questionnaires mailed		24 Years	Incident CHD, i.e., non-fatal myocardial infarction, CHD death, angiogram-confirmed angina pectoris, coronary artery bypass grafting (CABG), stents, and angioplasty.	High years of baseline rotating night shift work were associated with a significantly higher CHD risk in both cohorts
8.	Johnson et al. [30]	2020	Prospective cohort study	Female registered nurses	n = 116,429	To investigate associations between night shift work and six CVD biomarkers to determine if shift work affects levels of biomarkers that are precursors to CVD.	Recent night shift work, duration of rotating night shift work.	Questionnaires by mail.	Women with a history of cancer, myocardial infarction, or stroke at the time of blood collection, and missing values for variables of interest.	1996-2001	Total cholesterol, low-density lipoprotein cholesterol, high-density lipoprotein (HDL) cholesterol, triglycerides, C-reactive protein (CRP), and fibrinogen.	Suggestive evidence of adverse short-term and long-term effects of night shift work on select cardiovascular disease biomarkers.
9.	Gu et al. [14]	2016	Prospective cohort study	Female registered nurses	n = 74,862	To examine associations between rotating night shift work and all-cause, cardiovascular disease (CVD), and cancer mortality	Lifetime rotating night shift work: never, 1–2, 3–5, 6–9, 10–14, 15–19, 20–29, and ≥30 years	Questionnaire-based outcome; medical records, death certificates, National Death Index.	Women with pre-existing CVD (n = 2,444) or any cancer other than non-melanoma skin cancer in or before 1988	22 Years	All-cause mortality, CVD and cancer mortality, and mortality from specific CVD and cancer sites with >100 deaths	Confirmed associations of rotating night shift work and total and CVD (particularly IHD) mortality,
10.	Jankowiak et al. [35]	2016	Cross-sectional study	Residents of the City of Mainz	n = 10,475	To examine the association between exposure to current and cumulative night shift work and subclinical parameters of atherosclerosis.	Current and cumulative night shift work	Digital photo-plethysmography, volume plethysmography, Duplex ultrasound	Older than 64 years (n = 3753), did not provide any occupational information (n = 57), or stated to have never been employed in their lifetime (for at least one year in part-time);	-	Arterial stiffness, Vascular function (reactive hyperemia index (RHI)), intima media thickness (mm)	Night shift leads to detrimental changes in the atherosclerotic process.
11.	Kader et al. [15]	2022	Prospective cohort study	Healthcare professionals in Stockholm (HEROMA)	n = 60,873 (30,398 IHD cohort; 30,475 AF cohort)	To examine the effects of various aspects of night and shift work on the risk of incident IHD and AF in a prospective design.	Day shift, afternoon shift, night shift	IHD and AF diagnoses from National and Regional Patient Registers	Diagnosis of IHD or AF before the first employment day or in the past 12 months.	Eight years (2008-2016)	Incidence of IHD, AF	Night work is associated with a high risk of incident IHD but not AF; quick returns from afternoon shifts high IHD risk.
12.	Zhao et al. [4]	2022	Cross-sectional study	Female registered nurses (Hebei Province)	n = 121,903	To analyze the interaction between age and frequency of night shift on the hypertension prevalence	Duration of night shift (five-10 shifts, <10 shifts)	Self-measured blood pressure electronic sphygmomanometer	Who took sick leave, maternity leave, or went out for further study.	-	Hypertension	Interaction between specific age groups and night shift frequency on the prevalence of hypertension among female nurses.
13.	Yang et al. [45]	2022	Prospective cohort study	UK adults	n = 36,939	Investigate the association between current shift work and the risk of CMM in patients with hypertension	Various frequency and duration of shift work	Detailed questionnaires from the UK Biobank	Participants with missing data, or diagnosed coronary disease	2006-2010	Cardiometabolic disease, cardiometabolic mortality	Night shift work is associated with higher CMM risk in patients with hypertension.
14.	Ho et al. [19]	2021	Prospective cohort study	UK adults	n = 238,661	To study the association between shift work and incident and fatal cardiovascular disease (CVD), and to explore modifying and mediating factors.	Various frequency and duration of shift work	Questionnaire, interviews, baseline measurements at the UK Biobank.	Incomplete data from the questionnaire, those with chronic comorbidities	11 Years	Incident and fatal cardiovascular disease	Shift workers had a higher risk of incident and fatal CVD compared with non-shift workers, after adjusting for socio-economic and work-related factors.

Quality assessment was performed on studies using the assessment of multiple systematic reviews (AMSTAR 2) for systematic reviews (Table 2). Newcastle-Ottawa tool for cohort studies (Table 3) and Joanna Briggs Institute (JBI) tool for cross-sectional studies (Table 4).

**Table 2:** AMSTAR 2 questionnaire for systematic reviews and meta-analysis. +: yes, -: No, ±: partial yes, N/A: not applicable, AMSTAR: assessment of multiple systematic reviews. Reference: [47].

Study	AMSTAR 2 Questionnaire															
	AMSTAR 1	AMSTAR 2	AMSTAR 3	AMSTAR 4	AMSTAR 5	AMSTAR 6	AMSTAR 7	AMSTAR 8	AMSTAR 9	AMSTAR 10	AMSTAR 11	AMSTAR 12	AMSTAR 13	AMSTAR 14	AMSTAR 15	AMSTAR 16
Rosa et al. [2]	+	+	-	±	+	+	N/A	+	+	+	N/A	N/A	+	+	N/A	+
Cheng et al. [3]	+	-	-	+	+	+	-	+	+	+	+	+	+	+	+	+
Su et al. [13]	+	+	+	+	+	+	N/A	+	+	+	+	+	+	+	+	+
Gibson [33]	+	+	-	+	-	-	±	+	+	+	N/A	N/A	+	+	N/A	+
Sooriyaarachchi et al. [40]	+	+	-	+	+	+	-	+	+	+	+	+	+	+	+	+

**Table 3:** Newcastle-Ottawa scale for cohort studies. The symbol '*' denotes the score given to each criterion, therefore **: two points, ***: three points, and ****: four points. Reference: [48].

Studies	Selection	Comparability	Outcome	Total score
Vetter et al. [12]	***	**	***	8
Johnson et al. [30]	***	**	**	7
Gu et al. [14]	****	**	***	9
Kader et al. [15]	****	**	***	9
Yang et al. [45]	****	**	***	9
Ho et al. [19]	****	**	***	9

**Table 4:** Joanna Briggs Institute critical appraisal tool for analytical cross-sectional studies. Q*: *question, JBI: Joanna Briggs Institute. Reference: [49].

Study	JBI question								
	Q1	Q2	Q3	Q4	Q5	Q6	Q7	Q8	Q9
Rizza et al. [29]	Yes	Yes	Yes	Yes	Yes	No	Yes	Yes	Yes
Jankowiak et al. [35]	Yes	Yes	Yes	Yes	Yes	Yes	Yes	Yes	Yes
Zhao et al. [4]	Yes	Yes	Yes	Yes	Yes	Yes	Yes	Yes	Yes

Discussion

This review confirms, in many ways, the detrimental effect of shift work on the cardiovascular system of the human body. A number of studies [5,6,8,50] have previously attempted to solidify this connection, using at times overlapping parameters to gauge the desired outcome. 

The studies we include in our review strongly establish shift work, primarily through the disruption of the body’s circadian rhythm, and associated psychosocial behaviors adversely impact the cardiovascular system in a multitude of ways, which include but are not limited to myocardial infarction, coronary heart disease, ischemic heart disease, atherosclerosis, and hypertension. Shift work, night work in particular contributes to an increase in the risk of diabetes [7,39], metabolic syndrome [40-42], obesity [51], factors that are tightly linked to the development of cardiovascular disease. Shift workers are more likely to develop insulin resistance, increased adiposity, and changes in lipid and glucose metabolism, which makes them almost twice as likely [40] to develop metabolic syndrome. 

At a physiological level, it has been hypothesized that circadian disruption causes an increased secretion of stress hormones like catecholamines and cortisol [7,27,28] and a decrease in the sleep hormone melatonin [31]. Employees involved in night shifts have lower melatonin levels during night work, which was found to be connected to a decline in DNA repair capacity, indicating an increase in oxidative stress [33,34].

**Figure 2:**
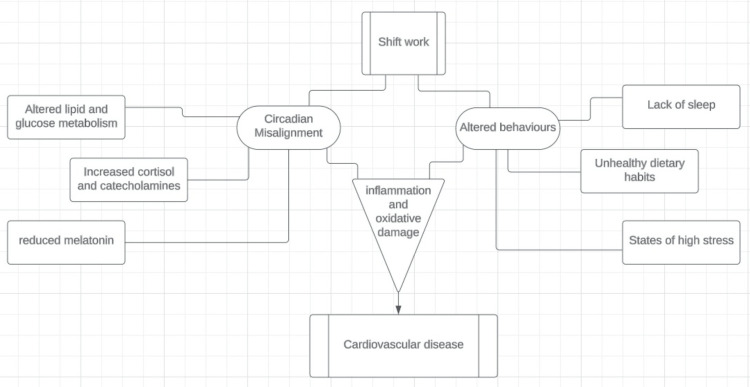
Multifactorial relationship between shiftwork and cardiovascular disease process. Image credits: Ayesha Hanif.

A summary of our results shows that there is an ample amount of data describing shift work and the negative influence it exerts on the cardiovascular system of those who do it for long periods of time (as briefly described in Figure 2). Our analysis is consistent with studies that have previously explored this association, and apart from some reporting mixed results [52-55], there seems to be a general consensus that shiftwork is a risk factor for CVD. Our review reflects this trend as other than one study that found no association between shift work and AF [15], all other studies showed a statistically significant association between shift work and some aspect of cardiac disease. The gaps, however, lie in the understanding of the pathological process involved at a micro level. There are several biomarkers to study oxidative stress and inflammation leading to cardiac or endothelial injury, but studies exploring these are limited with mixed results [30,53,56-59]. Additional biomarkers need to be studied and previous studies replicated to establish the validity of those results so they may be used to make better occupational policies.

There is also a need for more prevention-based studies, in a prospective study design preferably, to examine whether existing policies to improve occupational health are being implemented and whether they are successful in mitigating the negative effects of long shift hours. Some suggestions have previously been put forward that include timed naps [60], light-blocking glasses [61], and healthier meal options in work settings [62]. Education and awareness need to be provided to employers and management alike regarding the impact of shift work to encourage better scheduling practices. People in older age groups, those with prolonged and heavy shift work schedules, and those with comorbidities, for example, should be screened for cardiovascular changes early on [63]. Quick returns post-shift duty should be minimized [64]. We lack data regarding the effectiveness of such strategies [6,65]. A more consistent and coherent effort is required to tackle this growing problem. An overview of the studies based in healthcare settings shows that the majority consist of female nurses as their study population, while very little data is focused on junior doctors and fellows in training who regularly work schedules as part of their training.

There is a growing body of data regarding increasing amounts of exhaustion, burnout, and mental health deterioration faced by healthcare workers [1,66,67]. It is widely recognized that this leads to less patient safety and more workplace errors [68], but perhaps the lens needs to shift also to the cost of these rigorous schedules to the long-term health and safety of healthcare providers themselves. 

Strengths and Limitations

Our review’s biggest strength is that it gives a comprehensive and updated review of the recent research examining shift work's effect on the cardiovascular system in the past 10 years. Our study outcomes cover almost all major aspects of cardiovascular disease, such as hypertension, atherosclerosis, ischemic heart disease, arrhythmia, and metabolic syndrome. We also include recent studies that have attempted to shed light on the mechanisms behind these deleterious processes. Our review includes study populations across multiple professional settings. The findings in healthcare settings were consistent with those in non-healthcare settings. 

Despite most studies being good to high quality, they were susceptible to bias. Even after accounting and adjusting for various confounding factors, there could be a number of uncontrolled variables that could alter the results, such as intensity of shifts, patient load, and domestic and environmental stressors. Various studies also relied upon self-reported symptoms and employment/medical history, which could add discrepancies in the data. Another main limitation was the heterogeneity in shift work definition across different studies.

## Conclusions

Long-term shift work predisposes employees to a range of cardiometabolic diseases. These effects are increased with duration, frequency, and night shifts. Workers with comorbidities, increased age, and adiposity are at higher risk. More research is needed on biomarkers in disease processes and prevention strategies to curtail these damaging effects of shift work disrupted circadian rhythm. Implementation of better workplace policies needs to be ensured to prevent a growing workforce from the consequences of unhealthy scheduling practices. Thoughtfully constructed schedules that allow for a better work-life balance are essential to optimize the physical and mental well-being of physicians and nurses alike, which ultimately leads to better quality patient care as well.
