# Correction: Shifting Rhythms: A Systematic Review Exploring the Multifaceted Effects of Shift Work and Circadian Disruption on Employee Cardiovascular Health

**DOI:** 10.7759/cureus.c357

**Published:** 2025-10-13

**Authors:** Ayesha Hanif, Donatus K Okafor, Gitika Katyal, Gursharan Kaur, Hafsa Ashraf, Adiprasad Bodapati, Tuheen Sankar Nath

**Affiliations:** 1 Internal Medicine, Fatima Jinnah Medical University, Lahore, PAK; 2 Internal Medicine, California Institute of Behavioral Neurosciences & Psychology, Fairfield, USA; 3 Internal Medicine, Shalamar Medical and Dental College, Lahore, PAK; 4 Surgical Oncology, Tata Medical Center, Kolkata, IND

This article has been corrected to fix the following typos in the PRISMA diagram:

- 'Records screened' corrected from 241 to 243
- 'Reports sought for retrieval (n=37)' and 'Reports not retrieved (n=6)' added after omission
- 'Reports excluded' corrected from 14 to 17

